# Time–Frequency and Spectral Analysis of Welding Arc Sound for Automated SMAW Quality Classification

**DOI:** 10.3390/s26082357

**Published:** 2026-04-11

**Authors:** Alejandro García Rodríguez, Christian Camilo Barriga Castellanos, Jair Eduardo Rocha-Gonzalez, Everardo Bárcenas

**Affiliations:** 1 Facultad de Mecánica, Escuela Tecnológica Instituto Técnico Central, Calle 13 No. 16-74, Bogotá 111411, Colombia; 2 Departamento de Ingeniería Industrial, Universidad Nacional de Colombia, Carrera 45 No. 26-85, Bogotá 111321, Colombia; cbarrigac@unal.edu.co (C.C.B.C.); jerochag@unal.edu.co (J.E.R.-G.); 3 Facultad de Ingeniería, Universidad Nacional Autónoma de México (UNAM), Ciudad Universitaria, Ciudad de México 04510, Mexico; ismael.barcenas@ingenieria.unam.edu

**Keywords:** SMAW, acoustic monitoring, spectrogram, frequency spectrum, harmonic-to-noise ratio, machine learning, weld quality

## Abstract

This study investigates the feasibility of acoustic signal analysis for the assessment of weld bead quality in the shielded metal arc welding (SMAW) process. The work focuses on comparing time-domain acoustic signals and time–frequency spectrogram representations for the classification of welds as accepted or rejected according to standard welding inspection criteria. Two key acoustic descriptors, the fundamental frequency (F_0_) and the harmonics-to-noise ratio (HNR), were extracted and analyzed to evaluate statistical differences between the two weld quality classes. Statistical tests, including Anderson–Darling, Levene, ANOVA, and Kruskal–Wallis (α = 0.05), revealed significant differences between accepted and rejected welds. Accepted welds exhibited a bimodal HNR distribution associated with transient arc instability at the beginning and end of the bead, whereas rejected welds showed more uniform acoustic behavior throughout the process. Subsequently, the acoustic data were represented using both audio signals and spectrograms and used as inputs for ten supervised machine learning models, including Support Vector Classifier (SVC), Logistic Regression (LR), k-Nearest Neighbors (KNN), Decision Tree (DT), Random Forest (RF), Extra Trees (ET), Gradient Boosting (GB), and Naïve Bayes (NB). The results demonstrate that spectrogram-based representations significantly outperform time-domain signals, achieving accuracies of 0.95–0.96, ROC-AUC values above 0.95, and false positive and false negative rates below 6%. These findings indicate that, while scalar acoustic descriptors provide statistically significant insight into weld quality, time–frequency representations combined with machine learning enable a more robust and reliable framework for automated non-destructive evaluation, particularly in manual SMAW processes under realistic operating conditions.

## 1. Introduction

Artificial Intelligence (AI) and machine learning (ML) have become increasingly valuable in modern manufacturing due to their versatility and predictive capabilities [1,2,3]. Their ability to model non-linear relationships, classify complex patterns, and optimize process variables has transformed industrial quality control strategies [4,5]. Among the processes that can benefit most from intelligent monitoring, welding remains a foundational joining method in manufacturing, accounting for a significant share of metal joining operations in sectors such as automotive and structural fabrication [6]. In automotive body production, for example, welding dominates the joining methods used in modern car bodies [7]. This proportion reflects its central role in structural integrity, productivity, and safety across industries such as construction, automotive, and energy [8].

The quality of a welded joint depends on numerous variables, such as thermal gradients, material composition, and welding parameters, but also on the experience and manual ability of the operator. Human expertise directly affects bead geometry, penetration, and defect formation [9]. Yet, this factor is extremely difficult to quantify because it involves motor coordination, hand–eye stability, and even auditory cues perceived subconsciously by the welder [10]. Consequently, translating human experience into measurable, objective data remains an open research problem. Developing intelligent systems that can interpret these subtle sensory and acoustic patterns could enable standardized and reproducible evaluation of manual welding performance, integrating human expertise into data-driven production systems [11].

In recent years, several studies have explored the integration of ML and signal analysis to characterize and control welding processes [12]. Luo et al. [13] developed an acoustic emission-based monitoring system for pulsed laser welding of aluminum, employing a hybrid convolutional neural network (CNN)–long short-term memory network (LSTM) architecture trained on time–frequency representations of acoustic emission (AE) signals. Their approach achieved 99–100% classification accuracy in differentiating full, partial, and lack of penetration states, demonstrating the robustness of deep models for non-stationary signal interpretation.

Similarly, Tomcic et al. [14] analyzed pulsed laser welding of copper, varying laser power and feed rate. Using Fourier, Wavelet, and Speech-analysis techniques, they extracted acoustic features and applied Gaussian process regression (GPR) to estimate weld depth, obtaining root mean square error (RMSE) values between 42.5 µm and 48.4 µm. Their findings confirmed that acoustic emissions contain sufficient information to estimate penetration depth, although the approach requires high-frequency acquisition equipment and is limited to laser-based setups.

Other authors have expanded this line of research to arc-based processes. Rohe et al. [15] examined gas metal arc welding (GMAW) by converting captured arc sounds into spectrograms and Fourier-based spectrograms. Using CNN, they achieved 87–94% classification accuracy in detecting process anomalies, such as unstable metal transfer or shielding gas fluctuations. While promising, their model relied heavily on controlled laboratory conditions and did not evaluate robustness to ambient noise or operator variability.

Stemmer et al. [16] advanced toward multimodal defect detection in robotic laser welding, integrating audio and video inputs through unsupervised deep learning architectures (autoencoders and contrastive learning). Their multimodal system achieved an average area under the receiver operating characteristic (AUC-ROC) of 0.92 across eleven defect categories, demonstrating that combining sensory modalities enhances defect discrimination but also increases computational cost and data requirements.

In contrast, Chen et al. [17] employed real-time acoustic monitoring to detect cracks and keyhole pores in laser welding. Using Mel-frequency cepstral coefficients (MFCC) and various ML algorithms: CNN, Support Vectorial Machine (SVM), Gradient Boosting (GB), Logistic Regression (LR), and K-Nearest Neighbors (KNN). They reported 69–91% accuracy depending on the defect type. Their work highlighted that preprocessing and noise reduction are crucial for reliable classification, especially under variable operating conditions.

Nefedyev et al. [18] extended ML applications to multi-pass steel welds, integrating acoustic emission-based non-destructive testing (NDT) with clustering algorithms to recognize defect signatures in real time. Although quantitative metrics were not disclosed, the study demonstrated the feasibility of acoustic-based defect detection during live welding operations.

Finally, Kumar et al. [19] introduced a signal-processing approach using the continuous wavelet transform based on fast Fourier transform (CWT-FFT) for arc welding quality evaluation. Voltage and current signals were sampled at 100 kHz using a 500 MHz differential probe. The authors demonstrated that the dispersion of wavelet coefficients correlates with the welder’s skill level and shielding gas type, validating CWT as an effective tool for non-stationary signal analysis, though the study lacked machine learning-based classification.

Collectively, these studies confirm that acoustic and process signals contain valuable information for detecting defects, estimating penetration depth, and assessing process stability. Nonetheless, most of the existing research focuses on automated or laser-based systems, which operate under controlled conditions. By contrast, SMAW remains dominant in the fabrication of large-scale components, pipelines, and structural assemblies in environments characterized by noise, variability, and limited sensing infrastructure. In such settings, quantifying human performance and ability simultaneously remains a major challenge.

In industrial practice, weld beads vary considerably in length and deposition time depending on joint geometry, operator technique, and accessibility. These differences cause the corresponding acoustic signals to exhibit unequal durations and temporal resolutions, which complicates direct comparison and feature extraction. Preprocessing included trimming of low-amplitude noise segments and normalization of the audio signals. Transforming raw audio into image-based representations such as spectrograms or spectrograms offers an effective strategy to standardize signals of different lengths, enabling models to focus on intrinsic frequency-intensity patterns rather than temporal duration.

In this context, authors such as Noguez et al. [20] have demonstrated that the number of spectrogram images is a critical factor influencing model performance, yet datasets are often constrained by the limited availability of experimental data, particularly in welding-related studies where collecting consistent acoustic signals is time-consuming and equipment-dependent.

To address these gaps, the present study proposes a sound-based ML framework for evaluating SMAW weld bead quality. Audio recordings, which are simple to collect even in field environments, are transformed into time–frequency representations (spectrograms and spectrograms) that normalize signal duration and preserve temporal–spectral characteristics. A dataset of 400 weld bead recordings—comprising 200 acceptable and 200 defective samples, evaluated according to the American Welding Society, Welding Handbook Volume I: Welding Science and Technology, was used to train and compare ten ML models for binary classification.

Unlike automated welding processes, manual SMAW is characterized by significant operator-induced variability, including fluctuations in arc length, electrode manipulation, and heat input, which lead to highly nonstationary arc behavior and unstable metal transfer mechanisms. These conditions pose additional challenges for reliable acoustic-based monitoring, as the resulting signals exhibit stronger temporal variability and noise contamination. Therefore, despite the advances reported for automated welding systems, their direct transferability to manual SMAW remains limited, which highlights the specific need for the present study.

While previous studies have demonstrated the potential of acoustic and time–frequency analysis for automated and laser-based welding processes, the systematic application of these methodologies to manual shielded metal arc welding (SMAW) has received far less attention. Manual SMAW is intrinsically characterized by strong operator-dependent variability, continuous electrode consumption, arc instabilities, and high sensitivity to environmental disturbances. These factors introduce significant challenges for data-driven monitoring and limit the direct transferability of approaches developed for robotic or highly controlled welding systems.

In this context, the present work addresses this challenge by exploring the use of acoustic signal analysis and ML for the non-destructive assessment of weld quality in manual SMAW. This study is positioned at the intersection of human-centered manufacturing, acoustic monitoring, and intelligent quality control, with the aim of contributing to a better understanding of how arc sound patterns reflect process stability and weld integrity. By focusing on a widely used manual welding process under realistic operating conditions, this work seeks to extend acoustic-based intelligent monitoring beyond automated environments and toward field-relevant industrial applications.

## 2. Materials and Methods

This section describes the experimental setup, data acquisition procedure, and signal-processing pipeline adopted in this study. It details the welding conditions, acoustic recording system, preprocessing stages, and the construction of time–frequency representations used for feature extraction. Additionally, this section outlines the machine learning models, training strategy, and evaluation metrics employed for weld quality classification.

### 2.1. Materials Welding

Shielded metal arc welding was performed using AWS E6013 electrodes (1/8”) from three commercial brands: West Arco (Colombia), Lincoln Electric (Cleveland, OH, USA), and Ferretero (Colombia). All electrodes were new and extracted directly from sealed packages immediately prior to welding to ensure consistent moisture and coating conditions. The welding experiments were conducted using a Lincoln Electric Speedtec 200C inverter-based power source (Lincoln Electric, Cleveland, OH, USA).

The base material consisted of low-carbon A36 structural steel plates (local supplier, Bogotá, Colombia) with dimensions of 100 mm × 200 mm × 9.5 mm (3/8”). Prior to welding, all A36 steel plates were subjected to mechanical surface cleaning using a steel wire brush in order to remove mill scale, rust, and surface contaminants. After mechanical cleaning, the plates were degreased with acetone to eliminate residual oils and fine particles.

A total of 400 weld beads were produced and deposited on flat A36 steel plates: 200 classified as acceptable and 200 as defective according to the visual inspection criteria described in the Welding Handbook, Volume I: Welding Science and Technology, published by the American Welding Society, and Volume II: Welding Processes.

All weld beads were visually inspected by a certified welder following these handbook-based criteria. Welds exhibiting uniform bead morphology, stable arc behavior, and consistent material deposition were classified as accepted, while those showing pronounced geometric irregularities, surface discontinuities, or evidence of arc instability were classified as rejected.

The defective weld beads were intentionally generated by the welding personnel through controlled deviations from recommended operating conditions in order to reproduce realistic industrial defects. These deviations included the use of excessively high and low travel speeds, short and long electrode-to-plate distances, and welding current values intentionally set outside the nominal operating range for the selected electrodes. The combination of these variations promotes arc instability, irregular metal transfer, lack of fusion, excessive spatter, and surface discontinuities, which are typical causes of weld rejection in manual SMAW practice. This procedure ensured that the rejected class represents physically meaningful and reproducible defect conditions rather than artificially corrupted signals.

In particular, the evaluation was guided by the qualitative interpretation of arc stability and bead formation associated with current variations, as described in the “Effect of amperage on the welding process”. According to this reference, unstable arc behavior linked to inappropriate current levels is associated with irregular bead geometry, excessive spatter, lack of fusion, and surface porosity, which were used as rejection indicators in this study.

**Figure 1:**
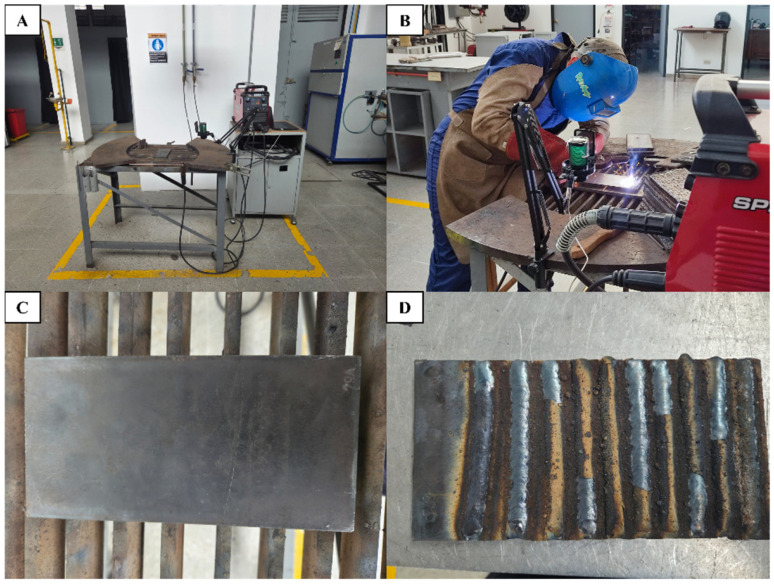
Experimental setup: (**A**) SMAW welding configuration with microphone; (**B**) welding and acoustic data collection process; (**C**) A36 plate before welding; (**D**) plate after bead deposition.

Figure 1 illustrates the experimental setup: (A) the SMAW welding station with microphone arrangement; (B) the welding and data acquisition process; (C) the A36 base plate before welding with the identification pattern; and (D) the completed plate containing all weld beads.

### 2.2. Welding Equipment

Weld beads were generated using a Lincoln Electric Speedtec 200C multiprocess inverter. The welding parameters are described in Table 1, depending on the bead type and electrode manufacturer. Voltage and arc length were manually adjusted by the operator to maintain stable arc conditions.

All welding parameters were recorded for traceability, and each bead was labeled with a unique identifier corresponding to its operator, current, and quality classification. Environmental noise was minimized during data acquisition. The duration of the welding process varied between approximately 15 and 45 s per weld bead due to the manual nature of SMAW. To ensure consistency in the analysis, fixed-length signal segments were extracted from each recording, allowing the models to focus on the acoustic characteristics of the welding arc rather than on variations in total welding time.

### 2.3. Acoustic Data Acquisition

Acoustic signals were collected using a MAONO DGM20S condenser microphone (MAONO Technology Co., Ltd., Shenzhen, China). with active noise cancellation, positioned 300 mm from the welding arc and oriented at 45° to the bead direction to avoid plasma interference. The microphone was connected to a digital audio interface, and recordings were captured using Audacity v2025 at a sampling rate of 44.1 kHz, stored in mp4 (mono, 16-bit) format.

Each welding session was recorded individually, and all files were trimmed and normalized to remove pre-arc and post-arc silence. A total of 400 audio samples were retained after quality verification for subsequent processing.

### 2.4. Signal Processing and Image Generation

The audio signals were converted into spectrogram images using Python 3.10 with the Librosa 0.11.0 and Matplotlib 3.10.8 libraries. For each recording, the short-time Fourier transform (STFT) was computed using the default configuration of librosa.stft function, which includes a Hann window, an FFT size (n_fft) of 2048 samples, a window length (win_length) of 2048 samples, and a hop length (hop_length) of 512 samples, corresponding to a 75% overlap between consecutive frames. Frame centering was enabled (center = True) and zero-padding was applied (pad_mode = “constant”). At the sampling rate of 44.1 kHz, this configuration provides a time resolution of approximately 11.6 ms and a frequency resolution of approximately 21.5 Hz. The STFT magnitude was mapped to a logarithmic (dB) color scale and visualized in 10 × 5 inch Matplotlib figures, which were saved as 300 dpi TIFF images with the axes removed in order to retain only the time–frequency information.

Subsequently, all spectrogram images were loaded and rescaled to a fixed resolution of 15 × 15 pixels using an image-resizing routine, and each image was flattened into a 225-dimensional feature vector, which was used as input to the ML models. Each spectrogram was categorized into one of the two classes:

- Class 0—Rejected welds (non-compliant according to Volume I and Volume II) were identified by the presence of defects such as lack of fusion, porosity, and irregular bead geometry, which are known to compromise the mechanical integrity of the joint.
- Class 1—Approved welds (acceptable according to Volume I and Volume II).

### 2.5. Statistical Analysis

The statistical comparison between the fundamental frequency (Fo) and harmonic-to-noise ratio (HNR) values of the different classes was carried out using both parametric and non-parametric approaches, depending on data normality and variance homogeneity.

The Fo was estimated as the inverse of the dominant period of the signal. Using an autocorrelation-based approach, Fo is defined as:

$$Fo=\frac{f_{s}}{\tau_{0}}$$

where $f_{s}$ is the sampling frequency and $\tau_{0}$ is the time lag corresponding to the maximum autocorrelation peak (excluding zero lag). This approach enables the identification of periodic components associated with arc stability during the welding process. The HNR was computed to quantify the balance between periodic and noise components in the acoustic signal. The calculation was based on an autocorrelation approach, defined as:

$$HRN=10\;{log}_{10}(\frac{R(\tau_{0})}{R(0)-R(\tau_{0})})$$

where $R(\tau_{0})$ corresponds to the autocorrelation at the fundamental period and R(0) represents the total signal energy.

The distribution of each variable was evaluated using the Anderson–Darling test (α = 0.05). This test was selected for its robustness with small-to-medium sample sizes and its sensitivity to deviations in the tails of the distribution. Homogeneity of variances was performed by Levene’s test (α = 0.05), which was used to verify the assumption of homoscedasticity across groups. Assumptions were violated, and the non-parametric Kruskal–Wallis test was applied as an alternative to ANOVA. Pairwise comparisons between classes were performed using the Bonferroni correction to adjust for multiple comparisons and reduce Type I error probability. All statistical tests were performed in Python (v3.10) using the SciPy and Pingouin libraries. Significant differences were reported at a confidence level of 95% (*p* < 0.05).

### 2.6. Machine Learning Models

Model evaluation was performed using stratified 10-fold cross-validation implemented through the StratifiedKFold routine from scikit-learn, with shuffling enabled and a fixed random seed (42) to ensure reproducibility. In each fold, approximately 80% of the data were used for training and the remaining 20% for validation, while preserving the original class distribution between accepted and rejected welds. Specifically, nine folds were used for training and onefold for validation in each iteration. The reported performance metrics correspond to the average results obtained on the validation subsets across all folds, ensuring that model evaluation was conducted on unseen data. Due to the limited size of the dataset, no separate hold-out test set was defined, and cross-validation was used as the primary estimate of generalization performance. To prevent data leakage, all preprocessing steps, including feature scaling, were implemented within scikit-learn pipeline objects, ensuring that transformations were fitted exclusively on the training data of each fold and then applied to the corresponding validation subset. Machine learning algorithms were implemented for classification: Support Vector Classifier (SVC), Logistic Regression (LR), Linear Support Vector Classifier (Linear SVC), Linear SVC with Calibrated Cross-Validation (L + C), Stochastic Gradient Descent Classifier with Calibration (S + C), K-Nearest Neighbors (KNN), Decision Tree (DT), Random Forest (RF), Extra Trees Classifier (ET), Gradient Boosting Classifier (GB), Gaussian Naïve Bayes (NB).

No data augmentation techniques were applied in this study. All machine learning models were trained and evaluated using only the original experimental spectrograms.

Hyperparameter optimization was performed using the Optuna library, varying the search space between 100 and 250 trials per model to converge toward the configuration yielding the highest accuracy and F1-score metrics. Each optimization run was limited to 14,400 s (4 h) of computation time, and convergence trends were monitored through historical optimization plots. Table 2 shows the hyperparameters optimized by each model of spectrum and spectrogram.

## 3. Results

This section presents the main experimental results obtained from the statistical analysis of acoustic descriptors and the machine learning classification models. It includes the performance comparison of the evaluated algorithms, the behavior of the extracted acoustic features, and the corresponding classification metrics used to assess weld quality.

**Figure 2:**
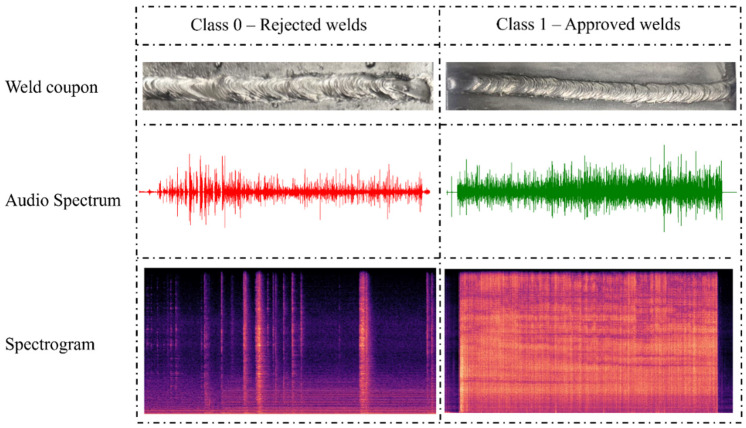
Representative images of rejected and accepted weld beads, along with their corresponding audio spectrum and short-time Fourier transform spectrograms for each manufactured specimen.

Figure 2 illustrates representative examples of accepted and rejected weld beads, showing their corresponding sound-wave spectra and spectrogram patterns. Analysis of the acoustic spectra revealed that rejected welds generally exhibited greater fluctuations in signal intensity, indicating a more heterogeneous and unstable arc behavior. In contrast, approved welds showed stronger and more uniform signal amplitudes, reflecting a steadier metal-transfer process.

These differences became even more pronounced in the spectrograms, where the joint representation of time, frequency, and intensity enhanced the discrimination between both classes. Rejected welds displayed abrupt tonal variations and irregular frequency-intensity distributions, typical of arc instability and inconsistent heat input. Conversely, approved welds exhibited a smoother profile, characterized by an initial and final attenuation of intensity and a more homogeneous central region, producing uniform color distributions across the spectrogram.

### 3.1. Frequency Distribution and Harmonic-to-Noise Ratio

**Figure 3:**
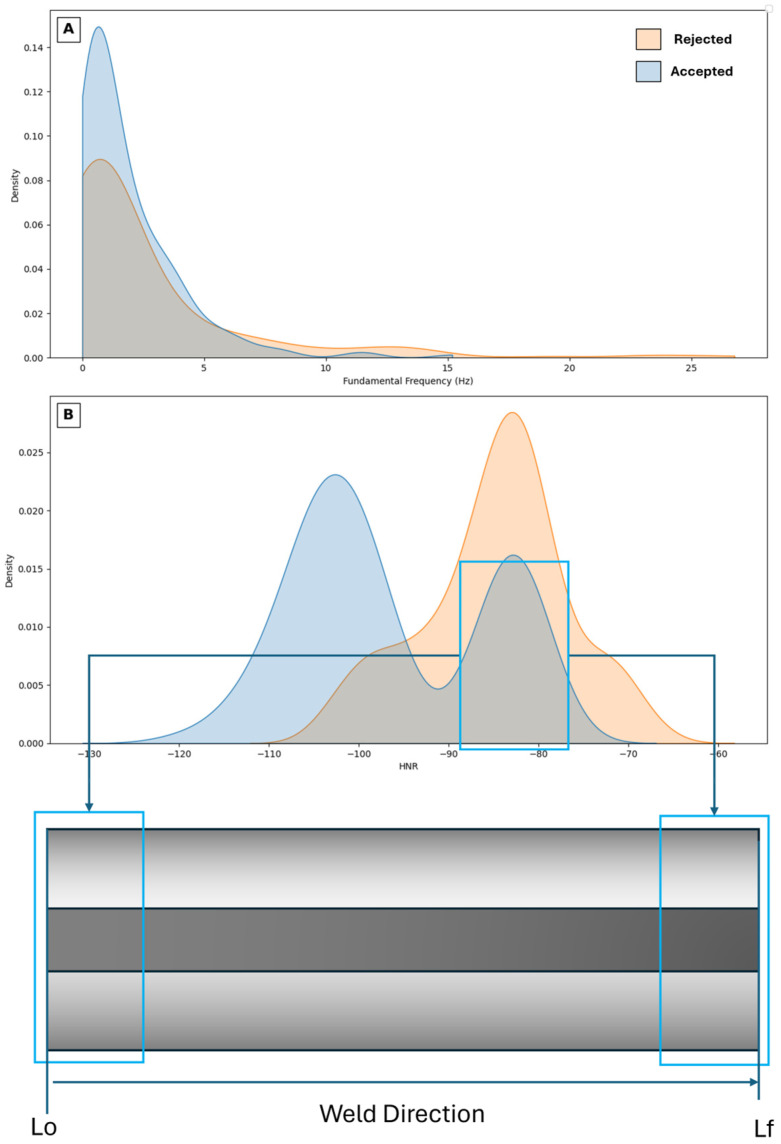
Distributions as a function of weld bead class: (**A**) Fo and (**B**) HNR and their relation between the initial point (Lo) and final point (Lf) of the weld.

Figure 3 shows the distributions of the Fo (Hz) and the HNR as a function of accepted and rejected weld beads. The Fo of both accepted and rejected welds was evaluated. The distribution corresponding to the accepted welds exhibits a shift toward lower frequencies and a narrower, more refined peak, which may be associated with the greater homogeneity of the acoustic signal. Conversely, the rejected welds display a broader peak centered at higher frequencies, indicating greater heterogeneity and instability in the sound emitted during welding.

Regarding the HNR, a clear bimodal pattern is observed for the accepted welds. The right-hand side of this distribution closely resembles that of the rejected welds. This dual behavior is attributed to the initial and final phases of the welding bead. When the electrode first establishes the arc, the cold base material absorbs heat, producing a distinct acoustic change. Similarly, at the end of the weld, the electrode length is significantly reduced, altering the sound emission and propagation along the bead. These transient variations stabilize during the central section of the weld, where the process reaches a thermoelectric equilibrium. Previous studies by Yusof et al. [21] and Szlapa et al. [22] reported similar trends, showing that the HNR rate evolves dynamically throughout the welding process, reflecting the transition between transient and steady-state acoustic regimes. Figure 3B presents a schematic illustration highlighting the origin of the second distribution of HNR values.

Table 3 summarizes the statistical tests and assumptions, showing that there are no significant overall differences between the accepted and rejected weld beads when analyzed as whole classes. For statistical analysis, the rejected weld class (Class 1) was further subdivided into two groups based on the distribution of the extracted acoustic features. Class 1-1 corresponds to rejected welds showing a clearly separated unimodal distribution with respect to accepted welds (Class 0), whereas Class 1-2 includes rejected welds with overlapping distributions. This subdivision was introduced to better interpret the statistical differences observed between groups. However, after separating the bimodal HNR distribution of the accepted welds into two subgroups—the low-HNR subgroup (Class 1-1) and the high-HNR subgroup (Class 1-2), a more detailed comparison was performed against the rejected welds (Class 0). The analysis revealed that the low-HNR subgroup (Class 1-1) exhibited statistically significant differences compared with the rejected welds (*p* = 7.7 × 10^−48^), indicating that this portion of the accepted class represents a distinct acoustic regime.

Conversely, the high-HNR subgroup (Class 1-2) showed no significant difference compared with the rejected welds (*p* = 0.52), suggesting that, acoustically, both share a similar harmonic-to-noise pattern during transient phases (i.e., start or end of the bead). These results reinforce the hypothesis that the dual HNR behavior in accepted welds originates from the transient acoustic states occurring at the beginning and end of the welding process, which acoustically resemble the rejected welds. For the Fo, although deviations from normality were detected (*p* < 0.001), the differences between classes were marginal (*p* ≈ 0.05), suggesting that frequency alone is not a strong discriminator between accepted and rejected welds.

The statistical comparison based on scalar acoustic descriptors, particularly the HNR, constitutes a first-level baseline for differentiating accepted and rejected welds. The statistically significant differences observed between the two classes confirm that the acoustic signal contains intrinsic information related to weld quality. The subsequent spectrogram-based ML analysis extends this baseline by incorporating the full time–frequency structure of the signal, allowing an automated and more robust characterization of the welding process beyond what can be achieved through scalar statistical descriptors alone.

### 3.2. Machine Learning Models-Sound Spectrum

Table 4 presents the classification performance of the evaluated machine learning models using audio spectrum representations. In general, the results show a moderate to high predictive capability, although consistently lower than those obtained using spectrogram-based features.

Among the evaluated models, Support Vector Classifier (SVC) and k-Nearest Neighbors (KNN) achieved the highest performance, with mean accuracies of 0.88 and 0.87, respectively, and ROC-AUC values up to 0.95. These results indicate that non-linear decision boundaries are beneficial when working with spectrum-based features, which may present complex but less structured relationships compared to spectrogram representations.

Ensemble methods such as Extra Trees (ET) and Random Forest (RF) also demonstrated competitive performance, with mean F1-scores around 0.85–0.87 and ROC-AUC values close to 0.93–0.94. However, their performance remains slightly inferior to that observed with spectrogram-based inputs, suggesting that the spectral representation alone does not fully capture the temporal dynamics of the welding process.

Linear models such as Logistic Regression (LR) and its calibrated variant (L + C) showed stable but comparatively lower performance, with mean accuracies between 0.84 and 0.87. This behavior suggests that the feature space derived from the audio spectrum is less linearly separable than that obtained from spectrogram representations.

The Naïve Bayes (NB) classifier exhibited the lowest performance, with a mean accuracy of 0.75 and ROC-AUC of 0.79, indicating that the independence assumption between features is not suitable for this type of representation, where spectral components are inherently correlated.

In terms of stability, most models presented standard deviation values between 0.03 and 0.06, indicating moderate variability across folds. Compared to spectrogram-based models, slightly higher variability is observed, particularly in Gradient Boosting (GB) and Decision Tree (DT), which may reflect sensitivity to variations in the spectral feature distribution.

**Figure 4:**
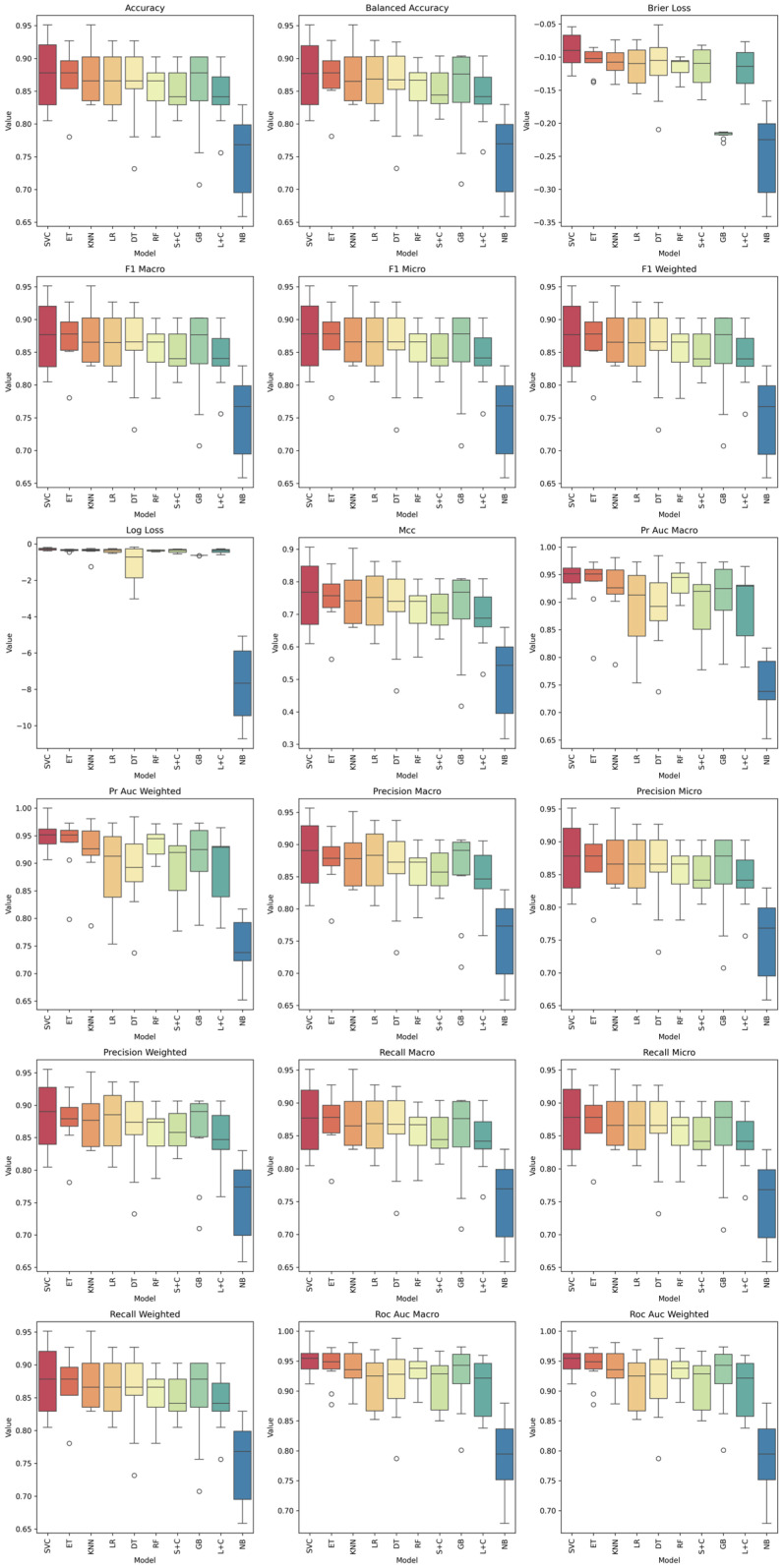
Comparative performance of the ML models for the binary weld quality classification task. Boxplots show the distribution of the evaluation metrics across 10-fold cross-validation for each model of sound spectrum, including accuracy, balanced accuracy, Brier loss, F1-macro, F1-micro, F1-weighted, log loss, Matthews correlation coefficient (MCC), PR-AUC macro, PR-AUC weighted, precision-macro, precision-micro, precision-weighted, recall-macro, recall-micro, recall-weighted, ROC-AUC macro, and ROC-AUC weighted.

Figure 4 shows the distribution of metrics of each model of spectrum sound. The highest values were observed for SVC, RF, and GB, with mean accuracies in the 0.85–0.88 range, demonstrating strong generalization and robustness. DT and KNN models also performed consistently but exhibited slightly larger interquartile ranges, indicating greater sensitivity to fold variations. In contrast, the MLP showed the widest spread and the lowest median accuracy, suggesting instability in training or possible overfitting due to the limited dataset size.

The F1-macro and F1-weighted metrics confirm that the models RF, GB and kernel methods like SVC maintain balanced performance between precision and recall across both classes (accepted/rejected). The SGD and MLP classifiers displayed lower F1 medians and higher dispersion, indicating class imbalance sensitivity. F1-macro between 0.88 and 0.92 for top models supports the discriminative capability of the acoustic features (HNR, Fo).

Ensemble methods (RF, GB) and SVC achieve the lowest Brier loss, evidencing well-calibrated probabilistic predictions. The log loss plots reinforce this assumption, with narrow ranges near optimal values. Models like MLP and SGD deviate significantly, suggesting poor probability calibration and higher uncertainty.

The MCC, a balanced metric even under class imbalance, follows a similar trend to accuracy: SVC, RF, and GB lead the ranking (MCC ≈ 0.85–0.90), confirming that these models produce coherent predictions across both classes. Lower MCC values in MLP indicate inconsistency in class prediction patterns.

ROC-AUC and PR-AUC (macro and weighted) show values exceeding 0.93 for the best-performing models (SVC, RF, GB), evidencing excellent separability between accepted and rejected welds based on acoustic parameters. Naïve Bayes maintains a stable but slightly lower performance (AUC ≈ 0.88), consistent with its assumption of feature independence. The MLP again appears as an outlier, showing a reduction of up to 10% in AUC compared with the top performers.

Precision and recall values follow the same pattern: ensemble and kernel-based models exhibit high central tendency and low dispersion, suggesting consistent classification of both accepted and rejected welds. The recall-weighted median above 0.90 for RF and SVC demonstrates that few defective welds were misclassified as acceptable, a crucial finding for real-world inspection systems.

**Figure 5:**
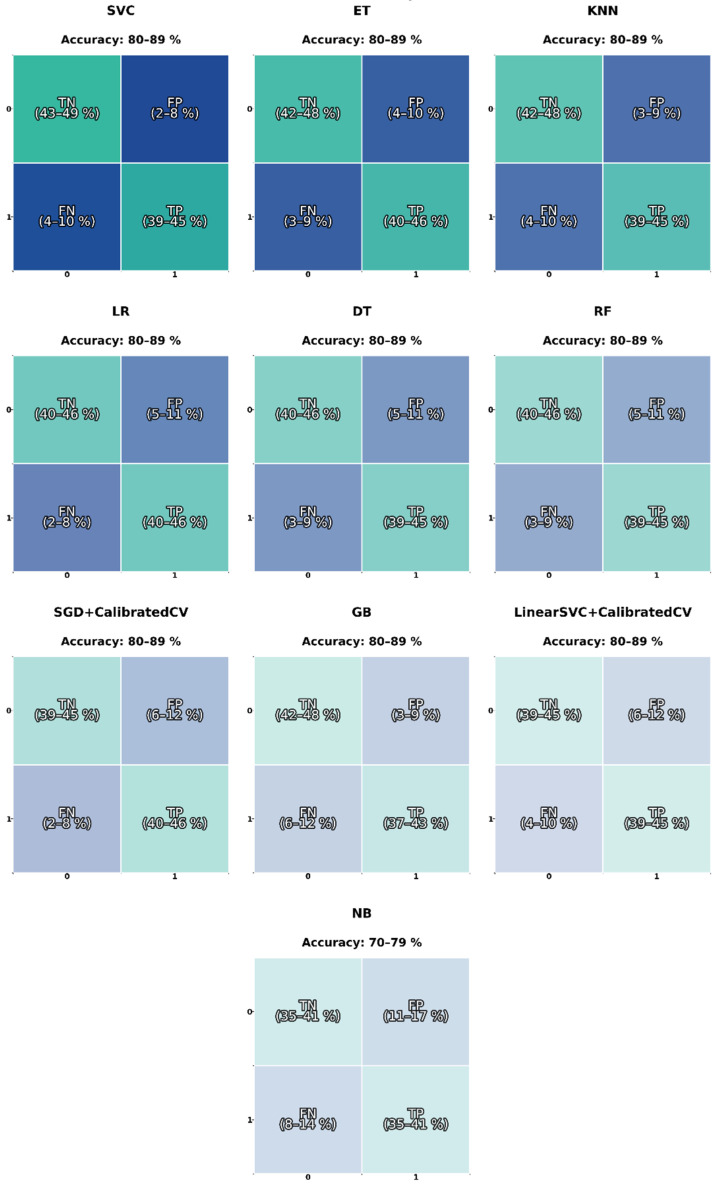
Confusion matrices of the ten ML models used for binary weld quality classification of the sound spectrum. Each matrix displays the proportion of true negatives (TN), false positives (FP), false negatives (FN), and true positives (TP) obtained through 10-fold cross-validation. The percentage ranges correspond to the minimum and maximum values observed across folds.

Figure 5 presents the average confusion matrices for all evaluated classifiers. Across most models, accuracies range between 80 and 89%, with balanced distributions of true positive (TP) and true negative (TN) rates. This symmetry indicates that the classifiers maintain balanced sensitivity and specificity, meaning they can identify both accepted (Class 1) and rejected (Class 0) welds without strong bias toward one class. SVC, RF, GB, ET, and KNN show the lowest FN and FP rates, generally under 10% for both classes. This behavior suggests a stable discriminative ability, allowing them to correctly identify most defective and acceptable welds. Their high TN and TP proportions imply strong generalization and robustness to intra-class variability in the acoustic signal.

These models achieve similar accuracy, with ranges between 80 and 89%, but with slightly higher FP dispersion (5–12%), implying less consistent probability calibration. DT and LR tend to slightly overpredict the “accepted” class, which could be due to class overlap in the feature space or weaker non-linear boundary modeling. However, their false negative (FN) rates remain low at 10%, which is desirable in a quality control context where missing defective welds is more critical than false alarms.

The Naïve Bayes classifier exhibits the lowest overall accuracy between 70 and 79%, mainly due to higher false positive (FP) and FN rates. This underperformance likely stems from its strong independence assumption between acoustic features, which is unrealistic in this context, as HNR, frequency, and spectral entropy are correlated during welding.

**Figure 6:**
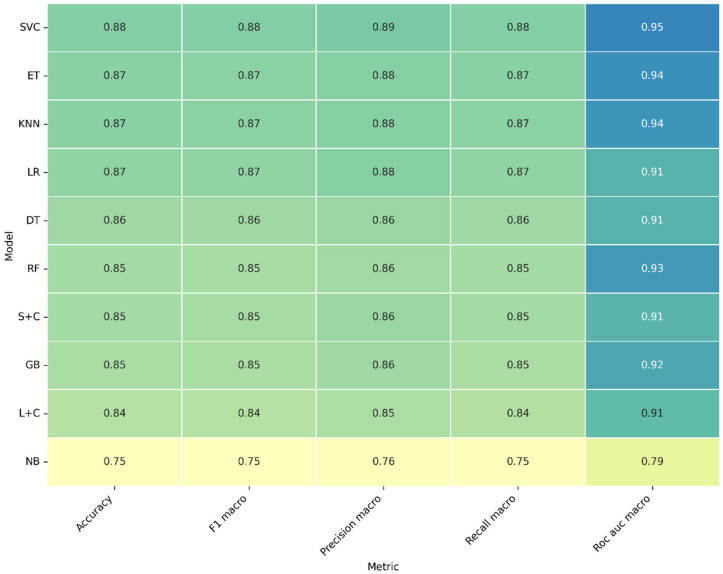
Mean performance metrics for the ten ML models used in weld quality classification of sound spectrum. The heatmap displays average values of accuracy, F1-macro, precision-macro, recall-macro, and ROC-AUC macro obtained from 10-fold cross-validation. Darker shades indicate higher performance.

Figure 6 displays the mean performance metrics for all evaluated classifiers. Most models achieved accuracy mean values above 0.85, confirming that the acoustic features provide high discriminative power for classifying weld quality. Only the NB classifier scored below 0.80 across all metrics, evidencing its limited capability to handle correlated acoustic variables. SVC outperforms the rest with: accuracy = 0.88, F1-macro = 0.88, ROC-AUC = 0.95. This superior behavior indicates that the SVC captured non-linear boundaries in the feature space, efficiently separating accepted and rejected welds. ET and KNN follow closely (AUC ≈ 0.94), showing strong robustness and generalization.

Ensemble approaches such as RF and GB maintain stable metrics (accuracy ≈ 0.85–0.86; AUC ≈ 0.92–0.93), confirming their capacity to model complex relationships between frequency and HNR without overfitting. Linear-based models (LR Linear SVC + Calibrated CV) perform slightly below kernel and ensemble methods, but still exhibit a reliable balance between precision and recall.

DT and SGD + Calibrated CV (S + C) display moderate scores, showing higher sensitivity to fold variation. Their simpler decision boundaries reduce their ability to capture subtle variations in acoustic patterns, particularly around transitional HNR regions between accepted and rejected welds.

The Naïve Bayes classifier consistently underperforms. This weakness is expected because NB assumes feature independence, an assumption violated in this dataset, where HNR and frequency are statistically correlated through the arc dynamics.

### 3.3. Machine Learning Models-Spectrogram

Table 5 summarises the classification performance of the evaluated machine learning models using spectrogram-based representations. Overall, the results indicate a consistently high level of predictive performance across most models, with mean accuracy values ranging from 0.84 to 0.95 and ROC-AUC values exceeding 0.91 in all cases.

Among the evaluated models, the Support Vector Classifier (SVC) and Logistic Regression (LR) exhibited the best overall performance. SVC achieved the highest mean accuracy (0.95) and F1-score (0.95), with a maximum accuracy of 1.00 and a ROC-AUC of 0.98, indicating excellent discriminative capability and robustness across folds. Similarly, LR and its calibrated version (L + C) demonstrated strong and stable performance, with mean accuracies of 0.94 and 0.93, respectively, and ROC-AUC values reaching up to 0.98–1.00.

Ensemble-based models such as Extra Trees (ET), Random Forest (RF), and Gradient Boosting (GB) also showed competitive performance, with mean F1-scores close to 0.89–0.90 and relatively low standard deviations, suggesting good generalization and stability. Notably, ET achieved a mean ROC-AUC of 0.96 with a low variability (std = 0.03), highlighting its effectiveness in capturing non-linear patterns from spectrogram features.

The k-Nearest Neighbors (KNN) model achieved comparable performance (mean accuracy = 0.91), although with slightly higher sensitivity to data variability. In contrast, the Naïve Bayes (NB) classifier presented the lowest performance among the evaluated models, with a mean accuracy of 0.84 and lower ROC-AUC values (0.91), which may be attributed to its assumption of feature independence, not fully satisfied in spectrogram-based representations.

The low standard deviation values observed across most models (generally below 0.05) indicate a stable classification behavior and consistent performance across cross-validation folds. Furthermore, the close agreement between accuracy, precision, recall, and F1-score suggests a balanced classification with no significant bias toward any class.

**Figure 7:**
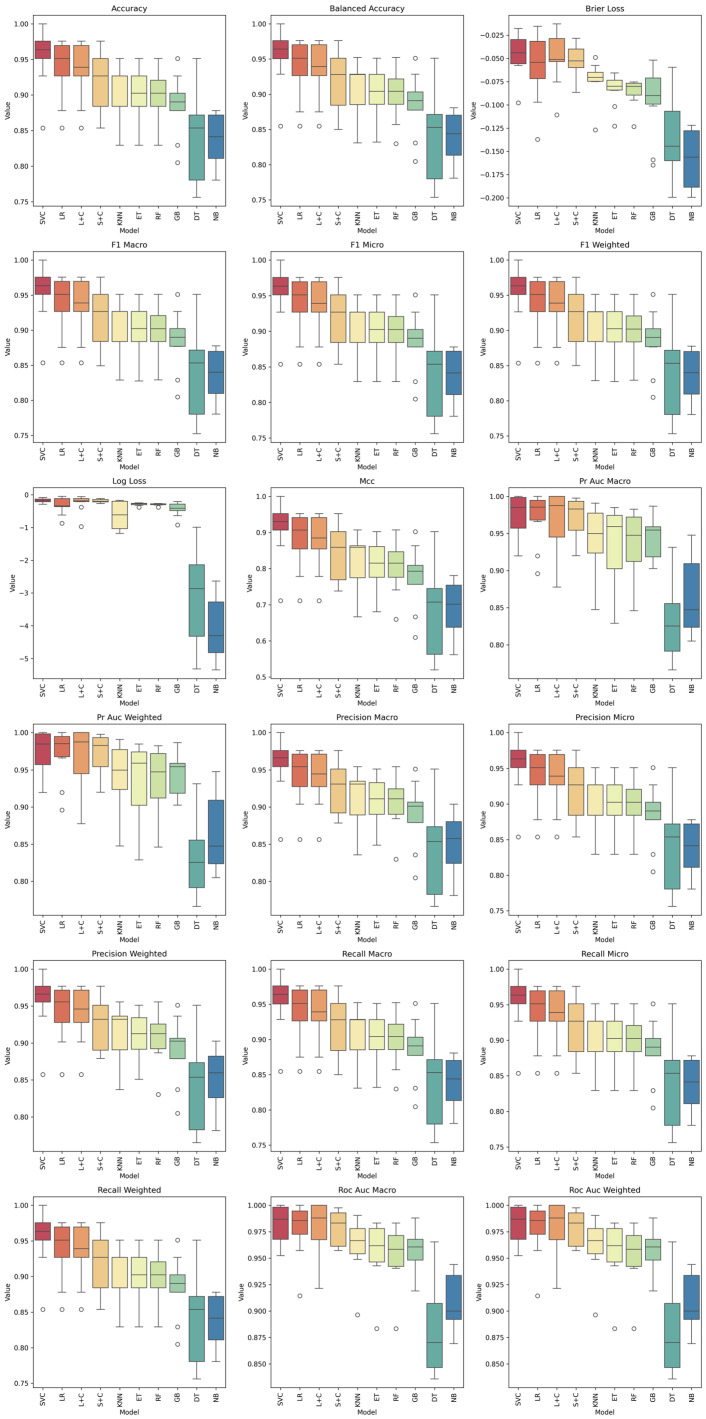
Comparative performance of the ML models for the binary weld quality classification task. Boxplots show the distribution of the evaluation metrics across 10-fold cross-validation for each model of sound spectrogram. accuracy, balanced accuracy, Brier loss, F1-macro, F1-micro, F1-weighted, log loss, Matthews correlation coefficient (MCC), PR-AUC macro, PR-AUC weighted, precision-macro, precision-micro, precision-weighted, recall-macro, recall-micro, recall-weighted, ROC-AUC macro, and ROC-AUC weighted.

Figure 7 illustrates the distribution of 18 classification metrics obtained from spectrogram-based features. All metrics show consistency among ensemble and kernel-based classifiers (SVC, RF, GB, ET), which exhibit narrow interquartile ranges and high median values, indicating robust generalization. The Naïve Bayes model remains an outlier across all metrics, evidencing its inability to capture the complex, correlated patterns inherent in spectrogram data.

SVC achieves the highest median accuracy (≈0.96) and balanced accuracy close to 0.95, outperforming all other models. RF and GB follow closely with accuracies between 0.92 and 0.94, suggesting that the spectral–temporal information encoded in the spectrograms provides rich discriminatory cues. The highly balanced accuracy values confirm that both classes (accepted/rejected welds) were equally represented and correctly classified, minimizing bias. The F1-macro, Precision, and Recall metrics confirm that top-performing models maintain consistent precision-recall balance across classes. The F1-macro values for SVC, RF, and GB exceed 0.93, indicating that the spectrogram-based features effectively capture both harmonic and noisy components relevant to weld quality.

The overlap between precision-macro and recall-macro distributions shows that FP and FN are balanced, a critical property for real-time inspection systems. All ensemble and kernel models achieve ROC-AUC and PR-AUC values above 0.95, demonstrating excellent separability between classes. The SVC reaches up to AUC = 0.98, indicating that the time–frequency representation of the acoustic signal enhances model sensitivity and specificity compared with scalar descriptors: mean HNR or Fo.

Low Brier and log loss values for SVC, RF, and GB confirm strong probabilistic calibration, meaning their predicted probabilities align closely with true class distributions. Matthews correlation coefficient (MCC) values above 0.9 reinforce the balanced performance across both categories.

Comparing these results with the models trained on scalar acoustic features, the spectrogram-based models show a clear performance improvement of approximately 7–10% in accuracy and F1, and 0.05 in ROC-AUC. This improvement validates that spectrograms preserve temporal dependencies and non-linear transitions in the welding sound, which are lost when using averaged parameters.

**Figure 8:**
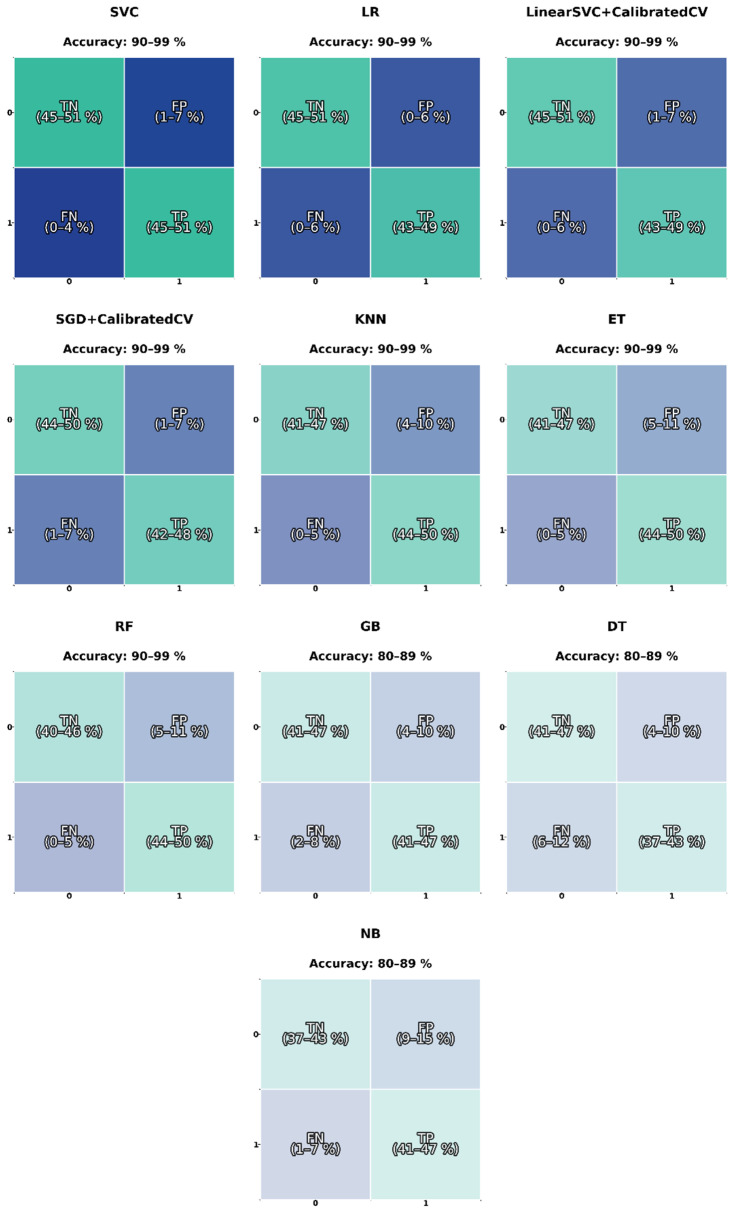
Confusion matrices of the ten ML models used for binary weld quality classification of the sound spectrogram. Each matrix displays the proportion of true negatives (TN), false positives (FP), false negatives (FN), and true positives (TP) obtained through 10-fold cross-validation. The percentage ranges correspond to the minimum and maximum values observed across folds.

Figure 8 shows the average confusion matrices for the models trained using acoustic spectrogram features. Each matrix represents the TP, TN, FP, and FN, averaged over ten cross-validation folds, along with the corresponding accuracy range.

Most models reached accuracy between 90% and 99%, demonstrating consistent and reliable classification performance across both accepted and rejected welds. The best results were obtained with the SVC, LR, and Linear SVC + Calibrated CV models, which displayed highly balanced predictions. These models achieved TP and TN rates of around 45–50%, while maintaining FP and FN below 6%, indicating that they can correctly distinguish between both classes with minimal bias.

Models such as SGD + Calibrated CV, KNN, and ET also performed well, though they exhibited slightly higher variability in FP and FN (up to about 10%). This suggests that they are somewhat more sensitive to variations in the acoustic signal across samples. The RF model maintained high TP rates between 44 and 50% but showed a small increase in FP, likely due to more aggressive decision boundaries that occasionally misclassified borderline signals.

The GB and DT classifiers had slightly lower accuracies (80–89%), mainly because of an increase in false negatives; some acceptable welds were misclassified as defective when their acoustic patterns were less defined. As expected, the Naïve Bayes model produced the weakest results, with higher FP (<15%) and FN (7%), reflecting its limitations when features are highly correlated and non-linear, as in spectrogram data.

These results confirm that using spectrogram representations significantly improves model performance compared to scalar features like Fo alone. The SVC, LR, and Linear SVC + Calibrated CV classifiers proved to be the most consistent and accurate, highlighting their robustness and ability to capture subtle harmonic and noise variations. These findings demonstrate that time–frequency acoustic analysis provides valuable information about arc stability and weld quality, supporting its potential use in real-time, non-destructive monitoring systems for welding processes.

**Figure 9:**
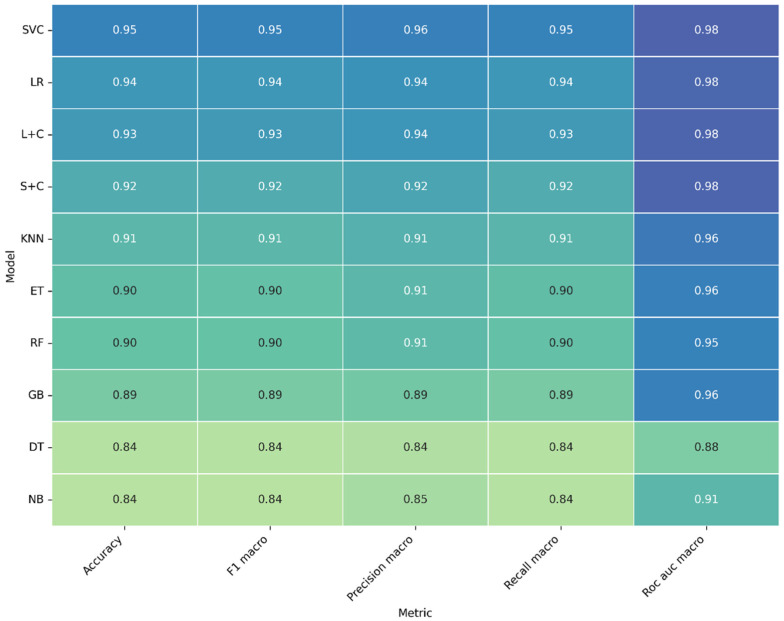
Mean performance metrics for the ten ML models used in weld quality classification of the sound spectrogram. The heatmap displays average values of accuracy, F1-macro, precision-macro, recall-macro, and ROC-AUC macro obtained from 10-fold cross-validation. Darker shades indicate higher performance.

Figure 9 summarizes the average performance metrics for the models trained with spectrogram features. Each cell represents the mean value obtained through 10-fold cross-validation for five key metrics: accuracy, F1-macro, precision-macro, recall-macro, and ROC-AUC macro.

All models achieved strong and consistent results, with most reaching AUC values above 0.95. The SVC stood out as the top performer, obtaining accuracy and F1 scores of 0.95–0.96 and an AUC of 0.98, confirming its excellent capacity to separate the two welding quality classes. LR and L + C models followed closely, both achieving nearly identical performance, showing that linear decision boundaries remain effective when properly calibrated.

Models such as S + C, KNN, and ET also maintained high reliability, while RF and GB performed slightly lower but still within excellent ranges. Only DT and NB fell below the top-performing group, with overall accuracies around 0.84, suggesting that simpler models struggle to capture the full complexity of spectrogram patterns.

The general trend indicates that spectrogram-based learning captures richer temporal and harmonic structures of the welding signal compared to scalar features. The small gap between accuracy, precision, and recall demonstrates balanced behavior, meaning that both accepted and rejected welds are correctly classified with minimal bias. These results confirm that spectrogram features provide the most discriminative representation of the welding process, enabling models like SVC and LR to achieve near-perfect classification. This reinforces the potential of acoustic spectrograms as a reliable non-destructive indicator of weld quality, suitable for implementation in real-time monitoring systems.

## 4. Discussion

This section discusses the results in relation to the physical welding process and the findings reported in previous studies. The implications of acoustic-based monitoring for manual SMAW are analyzed, including the influence of operator variability, signal stability, and the advantages and limitations of the proposed machine learning framework.

The results demonstrated that spectral and spectrogram-based acoustic information can effectively predict the quality of weld beads produced by the SMAW process. An analysis of the HNR distributions for accepted welds revealed a bimodal pattern, suggesting that the initial and final segments of the bead are acoustically distinct and can be identified through sound. Interestingly, the rejected welds exhibited HNR distributions similar to those of these critical regions, indicating that high HNR variability in these temporal windows may correspond to welds of lower quality.

These findings are promising, as they show that frequency and HNR distributions can serve as complementary, non-destructive indicators of bead quality. This is consistent with recent literature, where time–frequency descriptors have been proven to carry valuable diagnostic information. For instance, Yusof et al. [23] used synchro squeezed wavelet analysis of sound signals in laser welding and demonstrated that time–frequency representations effectively differentiate penetration conditions with high accuracy.

When the acoustic signals were converted into spectrograms and spectrogram images and used as input for ML models, classification accuracy exceeded 80% in most cases, with relatively low misclassification of rejected welds. Spectrograms yielded the best results, as they integrate time, frequency, and intensity information, thus enriching the input data for model training. This led to lower metric dispersion and higher overall precision.

A direct quantitative comparison between the discrimination capability provided by scalar acoustic descriptors (F_0_ and HNR) through statistical analysis and the performance of spectrogram-based machine learning models shows that the use of spectrogram features improves the effective classification accuracy by approximately 7–10% and increases the ROC-AUC. This improvement highlights the advantage of preserving the full time–frequency structure of the acoustic signal, which contains richer diagnostic information than scalar statistical descriptors alone.

The SVC and LR models displayed particularly balanced performance, with accuracy, F1, precision, and recall values closely aligned. False positive and false negative rates below 6% confirm that both classes were correctly discriminated against. From an NDT perspective, the false negative rate is the most critical metric, as it corresponds to defective welds that would be incorrectly accepted. An FN rate below approximately 6–10% implies that fewer than 6–10 defective welds out of every 100 inspected would remain undetected by the automated system. Such performance levels indicate a relatively low probability of missed defects and are consistent with the requirements of early-stage automated quality screening. In contrast, false positive errors mainly affect production efficiency by increasing rework or additional manual inspection of acceptable welds, rather than compromising structural safety. Since acceptable FN and FP tolerances strongly depend on the specific industrial sector, safety classification, and applicable standards, the proposed system is positioned as a decision-support or pre-screening tool rather than as a standalone final certification method. Moreover, the stability across folds indicates strong generalization capability, which aligns with results reported by Mattera et al. [24], who achieved consistent classification of acoustic weld signals through time–frequency representations and convolutional models.

These findings are consistent with recent advances in acoustic-based weld quality monitoring. Griffin et al. [25] demonstrated that short-time Fourier transform (STFT) analysis of acoustic emission signals can effectively identify imperfections produced by the metal active gas (MAG) process, linking spectral patterns with weld defects. In the field of laser welding, Luo et al. [13] used CNN–LSTM models fed with time–frequency AE features to monitor penetration depth, showing that spectrogram representations improve detection robustness. Likewise, Tomcic et al. [14] correlated AE signatures with weld depth, confirming that acoustic emission patterns reflect melt-pool dynamics. Together, these studies strengthen the evidence that acoustic signals are reliable proxies of weld quality when analyzed in the time–frequency domain.

The false negative rate (FN < 10%) obtained in this study meets the acceptance thresholds commonly considered in non-destructive inspection (NDI) standards, indicating that this approach is suitable for field deployment. Recent research supports these conclusions: Wolf et al. [26] demonstrated the feasibility of structure-borne sound-based in-process weld quality monitoring and Tomcic et al. [14] showed that data-driven MIG welding monitoring using ML achieves high reliability in detecting anomalies.

These studies highlight the growing industrial interest in “listening systems” that can automatically identify arc instabilities and signal quality deviations in real time. For industrial adaptation, further work is required to ensure domain adaptation, robust cross-validation, and noise resilience under varying conditions of torch geometry, joint configuration, and acoustic interference. Techniques such as transfer learning applied to acoustic spectrograms, as demonstrated by Bergström [27] in ultrasonic weld inspection, and multimodal fusion combining acoustics, vision, and thermal sensing, are promising strategies to increase robustness. Recent developments in multimodal monitoring networks [28] further validate the integration of spectrogram-based features for intelligent welding supervision.

This study confirms that acoustic spectrogram analysis combined with ML provides a powerful, non-destructive framework for SMAW weld quality assessment. By capturing the temporal and spectral fingerprints of the arc, this approach enables accurate classification of weld states and opens the door to intelligent, real-time monitoring systems capable of improving process stability, safety, and productivity in industrial welding environments. Moreover, this work represents the first step toward extending the study to a multiclass investigation, aiming to identify different types of defects in this welding technique.

Although stratified 10-fold cross-validation provides a robust estimate of model performance for moderate-sized datasets, the use of high-dimensional flattened spectrogram representations relative to the available number of samples may increase the risk of overfitting and optimistic performance estimation. In addition, the hyperparameter optimization process, which explores a large search space, may further accentuate this effect. While strict fold-wise preprocessing was applied to prevent data leakage, neither nested cross-validation nor a fully independent external test set was used in this study. Consequently, the reported performance values should be interpreted as optimistic estimates of generalization. Future work will therefore focus on validating the proposed approach using nested cross-validation and independent datasets acquired in different experimental campaigns and industrial environments.

An additional limitation of the present study is that a stratified robustness analysis with respect to electrode brand, welding current range, and bead length could not be performed, as the experimental campaign was conducted within a constrained set of consumables and operating conditions. Although this ensured high internal consistency of the dataset, it does not allow for a full assessment of the model’s robustness against variations in process parameters. In addition, the analysis was performed using full-length acoustic recordings of the weld beads rather than short sliding windows suitable for strict real-time implementation. While the reported results demonstrate strong offline classification performance, a dedicated segment-level evaluation is required to fully validate the suitability of the proposed approach for online monitoring. Future work will therefore focus on acquiring more diverse datasets covering multiple electrode brands, wider current ranges, and different bead geometries, as well as on implementing sliding-window spectrogram analysis to validate the generalization capability and real-time performance of the proposed framework under realistic industrial conditions.

An important limitation of the present study is that all welding experiments were performed by a single human operator under controlled laboratory acoustic conditions. In addition, only one base material and one joint configuration were investigated. While this experimental design ensured high internal consistency and repeatability of the acoustic measurements, it does not fully capture the variability encountered in real industrial environments, where multiple welders, different materials, joint geometries, background noise levels, and shop-floor disturbances may strongly influence the acoustic signatures. Consequently, the generalization of the proposed framework to fully industrial settings should be interpreted with caution. Future work will therefore focus on extending the experimental campaign to multiple welders, diverse materials, and joint types, and realistic shop-floor conditions in order to comprehensively validate the robustness and industrial applicability of the proposed approach.

## 5. Limitations and Future Works

The acoustic signals were recorded using a microphone with active noise cancellation enabled to reduce environmental background noise. Although the welding arc generates a dominant broadband acoustic signal, the potential influence of noise cancellation on the recorded signal cannot be completely excluded. Future work will include controlled experiments comparing recordings with and without noise cancellation, as well as the incorporation of additional acoustic descriptors such as fundamental frequency (F0) and harmonic-to-noise ratio (HNR).

In addition, although the density vs. HNR distribution reveals a visible trend differentiating accepted and rejected welds, the separation between classes is not complete and exhibits overlapping regions. This highlights a limitation of relying exclusively on low-dimensional scalar descriptors, which, despite their physical interpretability, may not fully capture the complexity of the acoustic behavior of the welding process. Consequently, future work should explore the integration of richer feature representations and advanced modeling approaches, including hybrid frameworks that combine statistical descriptors with time–frequency representations, as well as deep learning techniques capable of automatically extracting discriminative patterns from raw acoustic signals.

From an application perspective, although the developed machine learning models demonstrate strong classification performance, their integration into real industrial environments was not addressed in the present study. Future work will focus on the development of a complete implementation framework for real-time weld quality monitoring based on acoustic signals. This includes the design of an end-to-end system comprising acoustic data acquisition through industrial-grade sensors, real-time signal processing and feature extraction, and deployment of trained machine learning models for online classification of weld quality. In addition, the implementation of a decision-support interface for operators and quality inspectors will be explored, enabling immediate feedback during the welding process. Such a framework would facilitate the transition from offline analysis to practical non-destructive evaluation systems applicable in industrial SMAW operations.

## 6. Conclusions

The main contribution of this study lies in the comparative evaluation of time-domain and time–frequency acoustic representations for weld quality classification, demonstrating that spectrogram-based approaches significantly improve classification performance under realistic SMAW conditions.

This study confirmed that fundamental frequency (F_0_) and harmonics-to-noise ratio (HNR) provide reliable acoustic indicators for distinguishing accepted and rejected manual SMAW welds. The observed bimodal HNR behavior in acceptable welds highlights the acoustic sensitivity of arc ignition, steady-state, and extinction phases, demonstrating that sound-based monitoring can objectively capture process stability.

The use of STFT-based spectrogram representations combined with classical machine learning algorithms enabled robust weld quality classification, with Support Vector Classifier and Logistic Regression achieving the highest performance. These results validate that computationally efficient and interpretable models can successfully perform non-destructive weld quality assessment under manual SMAW conditions.

## Figures and Tables

**Table 1:** Experimental current–voltage conditions and intended welding regimes for the manual SMAW experiments.

Test ID	Welding Current (A)	Welding Voltage (V)	Intended Condition
T1	70	22	Low current (cold weld)
T2	85	24	Near-optimal condition
T3	100	26	Optimal condition
T4	115	28	High current
T5	130	30	Overcurrent
T6	90	25	Near-optimal condition
T7	110	27	High current
T8	125	29	Overcurrent
T9	75	23	Low current
T10	105	27	Nominal condition

**Table 2:** Hiperparameter per model optimized with best accuracy.

Model	Type	Key Characteristics	Advantages	Params Spectrum	Params Spectrogram
LR	Linear	Probabilistic linear model	Interpretable, fast	‘C’: 0.010254887747416 solver’: ‘liblinear’	‘C’: 0.01025488774741 solver’: ‘liblinear’
SVC	Kernel-based	Finds optimal separating hyperplane	Effective in high-dimensional spaces	‘C’: 8803.830228846728 ‘gamma’: 0.00244596888 ‘kernel’: ‘rbf’	‘C’: 8803.83022884672 ‘gamma’: 0.0024459688 Kernel’: ‘rbf’
RF	Ensemble	Multiple decision trees	Robust, reduces overfitting	‘n_estimators’: 68 ‘max_depth’: 48 ‘min_samples_split’: 20 ‘min_samples_leaf’: 6	‘n_estimators’: 68 ‘max_depth’: 48 ‘min_samples_split’: 20 ‘min_samples_leaf’: 6
GB	Ensemble	Sequential boosting	High accuracy	{‘n_estimators’: 439 ‘learning_rate’: 0.000271 ‘max_depth’: 36 ‘min_samples_split’: 20 ‘min_samples_leaf’: 10	{‘n_estimators’: 439 ‘learning_rate’:0.0002717 ‘max_depth’: 36 ‘min_samples_split’: 20 ‘min_samples_leaf’: 10
ET	Ensemble	Randomized trees	Fast, good generalization	{‘n_estimators’: 445 ‘max_depth’: 40 ‘min_samples_split’: 2 ‘min_samples_leaf’: 1	{‘n_estimators’: 445 ‘max_depth’: 40 ‘min_samples_split’: 2 ‘min_samples_leaf’: 1
KNN	Instance-based	Distance-based classification	Simple, no training phase	{‘n_neighbors’: 11 ‘weights’: ‘distance’ ‘metric’: ‘manhattan’	{‘n_neighbors’: 11 ‘weights’: ‘distance’ ‘metric’: ‘manhattan’
NB	Probabilistic	Assumes feature independence	Very fast, low data requirement	Default	Default
DT	Tree-based	Rule-based splits	Easy to interpret	‘max_depth’: 4 ‘min_samples_split’: 5 ‘min_samples_leaf’: 11	‘max_depth’: 4 ‘min_samples_split’: 5 ‘min_samples_leaf’: 11
LinearSVC + CalibratedCV	Linear + probabilistic calibration	Linear + probabilistic calibration	Linear + probabilistic calibration	‘C’: 0.00440982	‘C’: 0.004409824
SGD + CalibratedCV	Linear + probabilistic calibration	Linear + probabilistic calibration	Linear + probabilistic calibration	‘alpha’: 0.059435 ‘l1_ratio’: 0.69494	‘alpha’: 0.05943559 ‘l1_ratio’: 0.6949461

**Table 3:** Statistical summary of the normality, homoscedasticity, and ANOVA tests applied to the HNR and Fo of accepted and rejected weld beads. * indicates statistical significance at *p* < 0.05; ** indicates statistical significance at *p* < 0.01.

Variable	Normality	Homoscedasticity	Test (Kruskal–Wallis)	Anova
HRN	2.0 × 10^−5^ *	1.0 × 10^−5^ *	1.0 × 10^−5^ *	Class 1-1 vs. Class 0: 7.7 × 10^−48^ ** Class 1-2 vs. Class 0: 5.2 × 10^−1^ Class 1-1 vs. Class 1-1: 2.8 × 10^−34^ **
Frequency	1.0 × 10^−6^ *	1.22 × 10^−2^	5.08 × 10^−2^	----

**Table 4:** Summary of classification performance metrics for the evaluated machine learning models using audio spectrum representation.

Model	Accuracy				F1_Macro				Precision_Macro				Recall_Macro				Roc_Auc_Macro			
	Max	Mean	Min	Std	Max	Mean	Min	Std	Max	Mean	Min	Std	Max	Mean	Min	Std	Max	Mean	Min	Std
DT	0.93	0.86	0.73	0.061	0.93	0.86	0.73	0.061	0.94	0.86	0.73	0.062	0.93	0.86	0.73	0.06	0.99	0.91	0.79	0.059
ET	0.93	0.87	0.78	0.043	0.93	0.87	0.78	0.043	0.93	0.88	0.78	0.042	0.93	0.87	0.78	0.043	0.97	0.94	0.88	0.032
GB	0.9	0.85	0.71	0.068	0.9	0.85	0.71	0.069	0.91	0.86	0.71	0.069	0.9	0.85	0.71	0.068	0.97	0.92	0.8	0.055
KNN	0.95	0.87	0.83	0.041	0.95	0.87	0.83	0.041	0.95	0.88	0.83	0.041	0.95	0.87	0.83	0.041	0.98	0.94	0.88	0.033
L + C	0.9	0.84	0.76	0.042	0.9	0.84	0.76	0.042	0.91	0.85	0.76	0.044	0.9	0.84	0.76	0.042	0.96	0.91	0.84	0.047
LR	0.93	0.87	0.8	0.045	0.93	0.87	0.8	0.045	0.94	0.88	0.8	0.048	0.93	0.87	0.8	0.044	0.97	0.91	0.85	0.047
NB	0.83	0.75	0.66	0.062	0.83	0.75	0.66	0.063	0.83	0.76	0.66	0.062	0.83	0.75	0.66	0.063	0.88	0.79	0.68	0.064
RF	0.9	0.85	0.78	0.038	0.9	0.85	0.78	0.038	0.91	0.86	0.79	0.038	0.9	0.85	0.78	0.038	0.97	0.93	0.88	0.026
S + C	0.9	0.85	0.8	0.034	0.9	0.85	0.8	0.035	0.91	0.86	0.82	0.034	0.9	0.85	0.81	0.034	0.97	0.91	0.85	0.043
SVC	0.95	0.88	0.8	0.054	0.95	0.88	0.8	0.054	0.96	0.89	0.8	0.054	0.95	0.88	0.8	0.054	1	0.95	0.91	0.027

**Table 5:** Summary of classification performance metrics for the evaluated machine learning models using spectrogram-based representations.

Model	Accuracy				F1_Macro				Precision_Macro				Recall_Macro				Roc_Auc_Macro			
	Max	Mean	Min	Std	Max	Mean	Min	Std	Max	Mean	Min	Std	Max	Mean	Min	Std	Max	Mean	Min	Std
DT	0.95	0.84	0.76	0.066	0.95	0.84	0.75	0.067	0.95	0.84	0.77	0.065	0.95	0.84	0.75	0.067	0.97	0.88	0.84	0.044
ET	0.95	0.9	0.83	0.04	0.95	0.9	0.83	0.04	0.95	0.91	0.85	0.036	0.95	0.9	0.83	0.039	0.98	0.96	0.88	0.03
GB	0.95	0.89	0.8	0.043	0.95	0.89	0.8	0.043	0.95	0.89	0.8	0.043	0.95	0.89	0.8	0.043	0.99	0.96	0.92	0.023
KNN	0.95	0.91	0.83	0.038	0.95	0.91	0.83	0.038	0.95	0.91	0.84	0.038	0.95	0.91	0.83	0.038	0.99	0.96	0.9	0.027
L + C	0.98	0.93	0.85	0.042	0.98	0.93	0.85	0.042	0.98	0.94	0.86	0.038	0.98	0.93	0.85	0.042	1.00	0.98	0.92	0.026
LR	0.98	0.94	0.85	0.042	0.98	0.94	0.85	0.042	0.98	0.94	0.86	0.038	0.98	0.94	0.85	0.042	1.00	0.98	0.91	0.026
NB	0.88	0.84	0.78	0.035	0.88	0.84	0.78	0.035	0.9	0.85	0.78	0.04	0.88	0.84	0.78	0.035	0.94	0.91	0.87	0.027
RF	0.95	0.9	0.83	0.039	0.95	0.9	0.83	0.039	0.95	0.91	0.83	0.036	0.95	0.9	0.83	0.039	0.98	0.95	0.88	0.029
S + C	0.98	0.92	0.85	0.04	0.98	0.92	0.85	0.041	0.98	0.92	0.88	0.035	0.98	0.92	0.85	0.041	1.00	0.98	0.96	0.017
SVC	1.00	0.95	0.85	0.041	1.00	0.95	0.85	0.041	1.00	0.96	0.86	0.039	1.00	0.95	0.85	0.04	1.00	0.98	0.95	0.018

## Data Availability

The data that support the findings of this study are available from the corresponding author upon reasonable request. The dataset includes SMAW time–frequency spectrograms, and extracted descriptors (F_0_, HNR) used to train and evaluate the ML models.
